# Gain‐of‐function of progesterone receptor membrane component 2 ameliorates ischemic brain injury

**DOI:** 10.1111/cns.14122

**Published:** 2023-02-16

**Authors:** Chao Zhou, Taiyang Zhu, Wanyan Ni, Hui Zhou, Jiaxing Song, Miao Wang, Guoliang Jin, Yan Zhou, Jingjing Han, Fang Hua

**Affiliations:** ^1^ Institute of Neurological Diseases Xuzhou Medical University Xuzhou China; ^2^ Department of Neurology The Affiliated Hospital of Xuzhou Medical University Xuzhou China; ^3^ Department of Neurology Xinqiao Hospital and The Second Affiliated Hospital, Third Military Medical University Chongqing China; ^4^ Department of Geriatrics The Affiliated Hospital of Xuzhou Medical University Xuzhou China; ^5^ Department of Interdisciplinary Health Science College of Allied Health Science, Augusta University Augusta Georgia USA

**Keywords:** BBB leakage, neurobehavioral dysfunction, neuroinflammation, neuronal death, PGRMC2

## Abstract

**Objective:**

Progesterone receptor membrane component 2 (PGRMC2) belongs to the membrane‐associated progesterone receptor family, which regulates multiple pathophysiological processes. However, the role of PGRMC2 in ischemic stroke remains unexplored. The present study sought to determine the regulatory role of PGRMC2 in ischemic stroke.

**Methods:**

Male C57BL/6J mice were subjected to middle cerebral artery occlusion (MCAO). The protein expression level and localization of PGRMC2 were examined by western blotting and immunofluorescence staining. The gain‐of‐function ligand of PGRMC2 (CPAG‐1, 45 mg/kg) was intraperitoneally injected into sham/MCAO mice, and brain infarction, blood–brain barrier (BBB) leakage, and sensorimotor functions were evaluated by magnetic resonance imaging, brain water content, Evans blue extravasation, immunofluorescence staining, and neurobehavioral tests. The astrocyte and microglial activation, neuronal functions, and gene expression profiles were revealed by RNA sequencing, qPCR, western blotting, and immunofluorescence staining after surgery and CPAG‐1 treatment.

**Results:**

Progesterone receptor membrane component 2 was elevated in different brain cells after ischemic stroke. Intraperitoneal delivery of CPAG‐1 reduced infarct size, brain edema, BBB leakage, astrocyte and microglial activation, and neuronal death, and improved sensorimotor deficits after ischemic stroke.

**Conclusion:**

CPAG‐1 acts as a novel neuroprotective compound that could reduce neuropathologic damage and improve functional recovery after ischemic stroke.

## Graphical abstract

Progesterone receptor membrane component 2 (PGRMC2) was expressed in different neural cells in the setting of cerebral ischemia. The intraperitoneal injection of CPAG‐1, a gain‐of‐function ligand of PGRMC2, conferred resistance to BBB leakage, astrocyte activation, microglial activation, neuronal death, and disruption of neuroplasticity while promoting the recovery of sensorimotor dysfunctions after ischemic stroke.

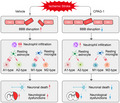

## 1 INTRODUCTION

Stroke is becoming one of the leading causes of mortality and long‐term disability across the world.^1^ Overall, 13.7 million people suffer from stroke each year, and over 70% of all stroke incidents are ischemic strokes.^2^ Although numerous achievements from benches to clinics have enriched our understanding of the pathophysiological mechanism of ischemic stroke, effective therapeutic approaches remain limited to thrombolysis and thrombectomy.^3^, ^4^ Thus, novel treatment paradigms for ischemic stroke are still in great need.

One of the pathological features of ischemic stroke is a sudden obstruction of the cerebral blood supply, which greatly reduces cerebral blood flow (CBF), increases neuronal death, and disrupts synaptic plasticity.^5^ The blood–brain barrier (BBB) is also disrupted in the core infarct and penumbra regions, allowing an increase in brain water and infiltration of peripheral immune cells, such as neutrophils and macrophages, to brain parenchyma from several minutes to hours after ischemic stroke and lasting for weeks.^6^, ^7^ Astrocyte and microglial cells are also initiated and migrate to the core and peri‐infarct regions.^8^, ^9^, ^10^, ^11^

Cumulative evidence has revealed that cerebral astrocyte and microglial cells are polarized to different phenotypes in the setting of ischemic stroke.^12^, ^13^ A1‐type astrocytes and M1‐type microglia are considered pro‐inflammatory phenotypes, while A2‐type astrocyte and M2‐type microglia are considered inflammatory‐resolving phenotypes.^12^, ^14^, ^15^ The infiltrated and endogenous inflammation largely hinders the recovery of sensorimotor dysfunctions.^6^, ^7^ The focus of current research is therefore the identification of neuronal‐protective and inflammatory‐resolving pharmacological targets for the treatment of ischemic stroke. One potential target is progesterone receptor membrane component 2 (PGRMC2).

Progesterone and its multiple receptors regulate diverse processes in the neurological system, such as neuroplasticity, neurogenesis, mood, and neuroinflammation.^16^, ^17^ PGRMC2 is a non‐classical progesterone receptor belonging to the b5‐like heme/steroid‐binding protein family.^18^ Galmozzi et al.^19^ have recently identified PGRMC2 as an intracellular heme chaperone and reported that brown fat‐selected deletion of PGRMC2 triggered mitochondrial defects. PGRMC2 was also confirmed as an inflammatory suppressor by sustaining the cytoplasmic localization of NFκB/p65 and restricting its transcriptional activity by interacting with GTPase activating protein binding protein 2 (G3BP2).^20^ Other studies have also shown that PGRMC2 acts as a suppressor of tumor migration by regulating cytochrome p450 activity^21^ and that the expression of PGRMC2 was downregulated in endometrial stroma cells of women with endometriosis.^22^ Currently, our understanding of the role of PGRMC2 in neurological diseases is limited. Intlekofer et al.^23^ found that PGRMC2 mRNA was widely expressed in neural tissue. We have previously reported that inhibition of PGRMC1 aggravated neuroinflammation and neuronal death after hypoxic–ischemic encephalopathy (HIE) in mice.^24^ However, the protein expression of PGRMC2 in different brain cells is unknown. The regulatory role of PGRMC2 in glial cell activation, BBB permeability, neuronal survival, and recovery of sensorimotor dysfunctions in the setting of ischemic stroke is still unclear.

In this study, male C57BL/6J mice were subjected to transit middle cerebral artery occlusion (MCAO), and the protein expression and localization of PGRMC2 were investigated. We synthesized CPAG‐1 (a gain‐of‐function ligand of PGRMC2) and measured the permeability properties of CPAG‐1. We also examined BBB leakage/permeability, sensorimotor dysfunctions, astrocyte activation, microglial activation, neuronal death/apoptosis, and synaptic plasticity after MCAO and CPAG‐1 treatment to explore the role of PGRMC2 in ischemic stroke.

## 2 MATERIALS AND METHODS

### 2.1 Animals

C57BL/6J mice were housed in groups of four per cage in a temperature‐ and humidity‐controlled animal facility with a 12 h light–dark cycle. Food and water were available ad libitum. All procedures using laboratory animals were approved by and conducted consistently with the guidelines of the Animal Care and Use Committees (IACUCs) of Xuzhou Medical University. All efforts were made to minimize animal suffering and the number of animals euthanized.

### 2.2 Murine model of ischemic stroke

Male adult C57BL/6J mice (7–8 weeks old, 20–25 g, Gempharmatech) were subjected to a 1 h intraluminal MCAO, followed by 1–7 day reperfusion, as described previously.^25^ Briefly, mice were anesthetized with 1.5%–3% isoflurane (Cat# R150‐22, RWD) in 100% oxygen. An incision was made in the middle of the neck, and a 2 cm length of a 6–0 rounded tip nylon suture (Cat# L2000, Jialing Biotech) was gently advanced from the external carotid artery to the internal carotid artery and further to the origin of the middle cerebral artery (MCA). After 1 h of MCA occlusion, reperfusion was performed by removing the suture. In sham‐operated mice, the same surgical procedure was performed, but with no suture insertion. Only male mice were included because stroke occurrence and severity can be affected by sex differences. The rectal temperature was controlled at 37.0 ± 0.5°C during surgery. The total number of animals used in this study and mortality rates are listed in Table S1.

### 2.3 CPAG‐1 preparation and treatment

The highly purified PGRMC2 gain‐of‐function ligand CPAG‐1 (HPLC > 99%, CAS#: 2094991‐62‐3) was synthesized, as described previously.^26^ The CPAG‐1 was dissolved in vehicle solution (dimethylsulfoxide [DMSO]:polyethylene glycol 40 [PEG40]:ethanol:phosphate buffered saline [PBS] = 2:3:1:4), as reported previously.^19^ Animals were randomly assigned to different groups using a lottery box. Male C57BL/6J mice were subjected to MCAO or a sham operation and then intraperitoneally injected with 45 mg/kg CPAG‐1 or vehicle solution at 6, 24, and 48 h after the onset of operation.

### 2.4 Measurement of CPAG‐1 concentration in plasma and brain tissue

The concentrations of CPAG‐1 in the brain parenchyma and plasma following MCAO were measured by injecting the mice intraperitoneally with 45 mg/kg of CPAG‐1 immediately after the onset of MCAO and again after 6, 12, and 24 h of reperfusion. The mice were deeply anesthetized, and the thoracic cavity was opened to expose the heart. The plasma and the contralateral/ipsilateral brain tissues were collected. The brain tissues were homogenized in ultra‐purified water, and CPAG‐1 was detected using liquid chromatography with tandem mass spectrometry (LC/MS), modified from our previous publication.^27^ Briefly, CPAG‐1 or internal standard (200 ng/mL of tolvaptan) was added to a 1.5 mL tube, and 50 μL of blank mouse plasma/brain sample suspension and 200 μL of acetonitrile were added to the tube. The mixed samples were vortexed for 10 min and centrifuged at 17,000 *g* for 10 min. The supernatant was evaporated to dryness and resuspended for LC/MC measurement. The calibration curve range of CPAG‐1 was 3.9–1000 ng/mL for plasma and brain homogenates.

### 2.5 Magnetic resonance imaging (MRI)

The brain infarct size was determined by 7.0 T MRI (Biospec 7 T/20 USR), as described in our previous publication.^28^ Briefly, mice were anesthetized with 1.5%–3% isoflurane and subjected to MRI scanning of the T2 sequence with the following parameters: thickness = 0.7 mm, TR/TE = 3000 ms/42.4 ms; matrix size = 256 × 256; field of view [FOV] = 2.3/2.0 cm; and FA = 90.0°. The T2‐weighted images were obtained and reconstructed using ParaVision 5.1 software. Brain edema was calculated based on MRI as follows: edema index = (ipsilateral volume – contralateral volume)/contralateral volume. The infarct volume was measured using ImageJ software and adjusted using the edema index.

### 2.6 Cerebral blood flow (CBF) measurement

The CBF changes were monitored using an RFLSI III Laser Speckle Imaging System (RWD), as described in our previous publications.^25^, ^29^ Briefly, experimental mice were anesthetized with 1.5%–3% isoflurane, and the skulls were exposed. The laser was placed 10 cm above the exposed skull surface. The CBF was measured 15 min before and after MCAO, 15 min after reperfusion, and 1 h after intraperitoneal injection. Regional CBF was calculated and analyzed using RWD LSCI software. The relative CBF was calculated as the ratio of the ipsilateral and contralateral hemispheres, which was then normalized to the mean value of the presurgery CBF baseline level for each animal.

### 2.7 Determination of brain water content

Brain water content was examined by the dry/wet method, as described in our previous publication.^24^ Briefly, the mouse was deeply anesthetized and decapitated 24 h after MCAO. The brain tissue was collected and separated into the contralateral and ipsilateral hemispheres and weighed (wet weight). The brain tissue was completely dried in a 100°C oven for 24 h and weighed again (dry weight). The brain water content was calculated by the following formula: water content (%) = 100 × (wet weight – dry weight)/wet weight.

### 2.8 Quantitation of Evans blue extravasation

The BBB leakage was evaluated by Evans blue extravasation, according to our previous publication.^30^ Briefly, at 23 h after MCAO/sham operation, the mice were intravenously injected with 200 μL 2% Evans blue in saline. At 1 h after injection, the mice were deeply anesthetized and transcardially perfused with saline to remove the Evans blue from blood vessels. Tissues from the contralateral and ipsilateral hemispheres were weighed and homogenized in 1 mL of N,N‐dimethylformamide solution. The resulting brain homogenates were incubated at 54°C for 48 h and then centrifuged at 14000× *g* for 20 min. The supernatant was collected, and the Evans blue levels in each hemisphere were determined using a plate reader at OD630. The Evans blue content was calculated from a standard curve.

### 2.9 Assessment of sensorimotor functions and neurological deficits

A battery of neurobehavioral tests was performed to assess sensorimotor functions after MCAO. On each testing day, all the neurobehavioral tests were conducted in the same order and duration to avoid the effects of the biological clock and light cycle on neurobehavioral results. We used the following sequence: corner test, cylinder test, adhesive tape removal test, foot fault test, and rotarod test.

#### 2.9.1 Corner test

Sensorimotor function was evaluated by the corner test, as described in a previous publication.^29^ Briefly, a small corner with a 30° angle was formed with two Plexiglas boards (30 × 20 × 1 cm). The experimental mice were placed halfway to the corner 10 times per day before surgery and again at 3, 5, and 7 days after surgery. Upon entering the corner, the mice reared forward and upward before making a U‐turn. The frequency of left turns and right turns was recorded. Turning movements that were not part of a rearing movement were not recorded.

#### 2.9.2 Cylinder test

The cylinder test was conducted to evaluate the forepaw sensorimotor functions before and 3, 5, and 7 days after surgery as described previously.^31^ Briefly, experimental mice were put into a Plexiglas cylinder (15 cm tall × 9 cm diameter), and a video camera was positioned above the cylinder to record for 10 min. The number of left, right, or both forepaw uses were counted when the mice first contacted the cylinder wall. The forepaw use asymmetry was calculated using the following formula: contralateral paw use (%) = 100 × right / (left + right + both).

#### 2.9.3 Adhesive tape removal test

Forepaw sensorimotor function was also evaluated by the adhesive tape removal test as described previously.^25^, ^29^ Briefly, experimental mice were placed in a transparent Plexiglas cylinder (30 cm tall × 20 cm diameter) for a 60 s habituation period before testing. A piece of adhesive tape (0.3 cm × 0.4 cm) was then placed on the hairless part of the left forepaw. The times when the adhesive tape was first touched (time to touch) and then completely removed from the forepaw (time to remove) were recorded. Three trials per day were conducted for 3 consecutive days before surgery and again at 3, 5, and 7 days after the operation. The maximum time between touch and removal was recorded as 120 s if the mouse did not touch or remove the adhesive tape.

#### 2.9.4 Foot fault test

Forepaw and hindpaw sensorimotor functions were examined by the foot fault test as described previously.^25^, ^32^ The mice were allowed to walk freely on a metal grid surface for 3 min, and a foot fault was counted when the forepaw or hindpaw fell or slipped between the wires. One trial per day was conducted 1 day before surgery and again 3, 5, and 7 days after the operation. Data were expressed as the percentage of error steps to the total moving steps of the contralateral forepaw/hindpaw.

#### 2.9.5 Rotarod test

Sensorimotor function was evaluated by the rotarod test as described previously.^29^, ^32^ Briefly, experimental mice were placed into a rotating drum (ZH‐600B, Anhui Zhenghua Biologic Apparatus Facilities) accelerating from 5 to 40 rpm within 5 min. Each mouse was examined 3–4 times/day for 3 consecutive days before and 3, 5, and 7 days after the operation, with a 5 min interval between each trial. The mean times of 3–4 trials each day on the rod (latency to fall) before surgery (baseline) and at selected time points after the operation were recorded.

#### 2.9.6 Neurological deficits

Neurological deficits were examined and scored on a 5‐point scale 3 days after MCAO, as previously described,^33^ using the following scores: 0, no observable neurological deficits (normal); 1, failure to extend right forepaw (mild); 2, circling to the contralateral side (moderate); 3, falling to the right (severe); 4, mice could not walk spontaneously; and 5, depressed level of consciousness (very severe). All stroke outcome assessments were performed in a blinded manner.

### 2.10 RNA‐sequencing

At 3 days after MCAO/sham operation and CPAG‐1/vehicle treatment, brain tissue from the ipsilateral hemisphere was collected, and the total mRNA was extracted using Trizol (ThermoFisher, #15596018), as described previously.^29^ The RNA quality and concentration were examined with a NanoDrop ND‐1000 instrument (NanoDrop), and the integrity of the RNA was assessed with a Bioanalyzer 2100 (Agilent). The RNA libraries were sequenced on the Illumina NovaseqTM 6000 platform by LC Bio‐Technology Co., Ltd. Clean data were obtained by filtering out unqualified sequences (sequencing adapters, low‐quality sequences, etc.). Hisat2 was used for reference genome alignment. The genome‐annotated protein‐coding genes (mRNA) were counted and analyzed as their Fragments Per Kilobase Million (FPKM) values to evaluate differentially expressed genes in different experimental groups.

Bioinformatics analysis was performed using the OmicStudio tools at https://www.omicstudio.cn/tool. The volcano map (ggplot2, OmicStudioClassic, and OmicStudioKits packages), heatmap (pheatmap package), and gene set enrichment analysis (GSEA; clusterProfiler and enrichplot packages) were drawn and analyzed using the R project (version 4.1.3) on the OmicStudio platform (https://www.omicstudio.cn/tool).

### 2.11 Immunofluorescence (IF) staining

The mice were deeply anesthetized and transcardially perfused with 30 mL of saline, followed by 30 mL of 4% paraformaldehyde. The brains were dissected, post‐fixed in 4% paraformaldehyde overnight at 4°C, and then immersed in 30% sucrose in 0.1 M phosphate buffer for another 2 days. When the tissues had completely dropped to the bottom of the 30% sucrose solution, the brains were cut into 25 μm coronal sections using a microtome (CM1950, Leica), and the brain sections were preserved in cryoprotectant at −20°C until further use.

Immunofluorescence staining was performed as previously described.^34^, ^35^ Briefly, brain sections were washed 3 times for 5 min with PBS in a 24‐well plate, permeabilized once for 20 min with 1% PBST (1% Triton‐× 100 in PBS) and washed twice for 5 min with 0.3% PBST. The free‐floating sections were then blocked with 5% normal donkey serum in 0.3% PBST for 1 h at room temperature. The mouse brain sections were then incubated with the appropriate primary antibodies (diluted in 0.3% PBST), followed by secondary antibodies. The primary antibodies used in this study, their corresponding dilution factors, and the vendor information are listed in the Table S2. Images were captured using a confocal microscope (A1R, Nikon) or a fluorescence scanner (Pannoramic MIDI, 3DHISTECH). The immunostained area of GFAP Iba‐1 and the numbers of immunopositive NeuN, TUNEL, and Neutrophil cells were processed for analyses with Image J software. Three randomly selected microscope fields from 3 consecutive sections (Bregma = 0.5 mm) in the cerebral cortex and striatum were analyzed for each brain.

### 2.12 Fluoro‐Jade B (FJB) fluorescence staining

The degenerated neurons after MCAO were examined by FJB (#TR‐150‐FJB, Biosensis) staining, as previously described.^24^ Briefly, brain sections (25 μm thick, bregma = 0.6 mm) were mounted on gelatin‐coated slides and dried overnight at room temperature. After rehydration and permeabilization, the slices were immersed in 0.06% potassium permanganate solution for 10 min to reduce background fluorescence. The slices were then incubated in 0.0002% FJB solution in the dark for 10 min, rinsed, dried in the dark, immersed in xylene, and cover‐slipped with a nonaqueous mounting medium. Images were obtained using a fluorescence scanner (Pannoramic MIDI, 3DHISTECH), and the FJB‐positive cells in the cortex and striatum were counted using Image J software.

### 2.13 Western blotting

Western blotting was performed as previously described.^36^ Total protein was extracted from the ipsilateral hemisphere, separated on 10% and 12.5% SDS–PAGE gels, and transferred to 0.45 μm polyvinylidene difluoride (PVDF) membranes. After blocking with 5% non‐fat dried milk in 0.1% TBST, the membranes were incubated with primary antibodies for PGRMC2, GFAP, Iba‐1, CD68, CD16/32, CD206, PSD‐95, synaptophysin, β‐actin, or GAPDH and incubated at 4°C overnight in a shaker. After three 5 min washes with 0.1% TBST, the PVDF membranes were incubated with secondary antibodies for 2 h at room temperature. The blots were washed three times with 0.1% TBST, and the proteins were detected using a gel imaging system (ChemiDoc XRS+, Bio‐Rad) and analyzed using Image J software. The protein expression levels were normalized to β‐actin or GAPDH. Primary antibody dilution factors and vendor information for the western blotting and IF staining protocols are listed in the Table S1.

### 2.14 Quantitative PCR

Total RNA was extracted from the ipsilateral hemisphere of the mouse brains using Trizol (Invitrogen). The quantitative real‐time reverse transcriptase‐polymerase chain reaction (RT‐PCR) was carried out using an ABScript III RT Master cDNA Synthesis Kit (#RK20429, ABclonal), TB Green® Premix Ex Taq™ II (#RR820A, TaKaRa), and a Bio‐Rad CFX Connect Thermocycler, according to published protocols.^24^, ^25^ Specific primers used for the PCR reaction in this study are listed in the Table S2. The relative mRNA expression was normalized to β‐actin RNA levels. The PCR experiments were repeated 3 times using separate mouse brain samples.

### 2.15 Statistical analyses

The normality of all data in this study was evaluated using the Shapiro–Wilk test. All data in this study are expressed as mean ± SD or mean ± range as dots and analyzed with GraphPad Prism 9 (GraphPad Software). For data that met the Shapiro–Wilk normality test and Brown–Forsythe homogeneity of variance test of multiple‐group comparisons, one‐way or two‐way ANOVA was used, followed by Tukey's post hoc test. The Welch ANOVA test and Dunnett T3 post hoc tests were used when the variances were heterogeneous. The Kruskal–Wallis test was used when the data distribution did not meet normal Gaussian distribution. A two‐tailed *t*‐test was used for a two‐group comparison. Mann–Wilcoxon rank sum test was used when the data were ordinal categorical variables. A value of *p* ≤ 0.05 was considered statistically significant. *Q* value (*p*‐value adjusted using an optimized FDR approach) was used for RNA‐sequencing data analysis and *q* ≤ 0.05 was considered statistically significant.

## 3 RESULTS

### 3.1 The protein expression of PGRMC2 is elevated in different brain cells after ischemic stroke

**FIGURE 1:**
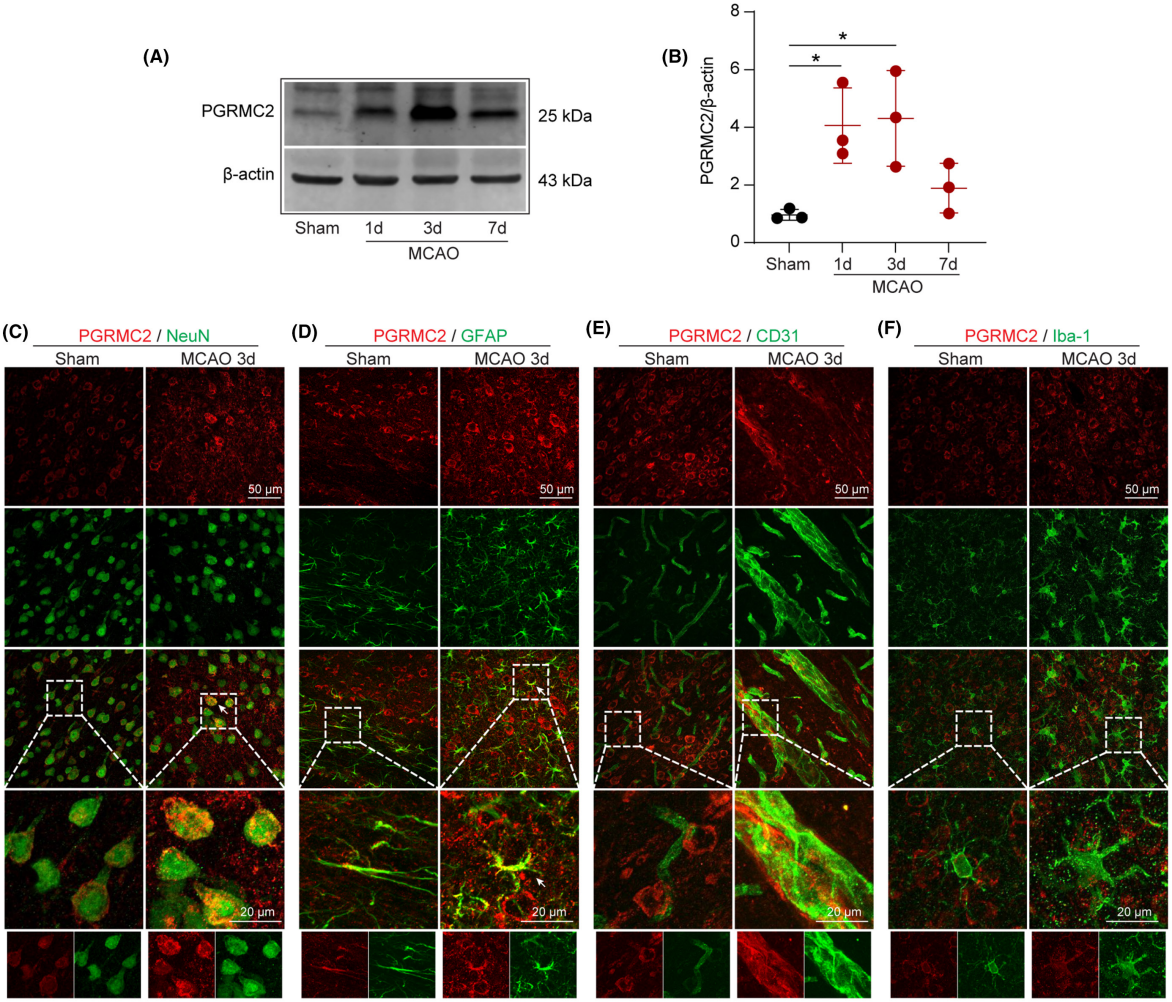
The expression of PGRMC2 was elevated in different brain cells in mice after ischemic stroke. Male C57BL/6J mice were subjected to sham or MCAO operations, and the protein expression of PGRMC2 was evaluated by western blotting at 1, 3, and 7 days after surgery. (A) Representative western blotting images of PGRMC2 in the mouse cerebral cortex at 1, 3, and 7 days after MCAO. (B) Quantitative analysis of PGRMC2 relative protein expression at 1, 3, and 7 days after MCAO (*n* = 3/group, one‐way ANOVA, and Tukey's post hoc test). The cell localization of PGRMC2 was evaluated 3 days after MCAO by dual‐immunofluorescence staining. Data are expressed as mean ± SD. *p < 0.05 versus sham group. (C‐F) Representative images of dual‐immunofluorescence staining of PGRMC2 (red) with NeuN (neuron marker, green), GFAP (astrocyte marker, green), CD31 (brain vessel marker, green), and Iba‐1 (microglia marker, green).

Male C57BL/6J mice were subjected to MCAO, followed by 1, 3, and 7 days of reperfusion (animals used and mortality in this study are listed in Table S3), and protein expression of PGRMC2 was determined by western blotting. The protein expression level of PGRMC2 was significantly higher in MCAO mice at 1 and 3 days after the operation than in the sham controls (Figure 1A,B). Further investigation of the localization of PGRMC2 in different brain cells 3 days after MCAO by dual‐immunostaining of PGRMC2 with NeuN (a neuronal nuclear marker), GFAP (an astrocyte marker), CD31 (an endothelial cell marker), and Iba‐1 (a microglia marker) revealed co‐staining of PGRMC2 with NeuN, GFAP, and Iba‐1 under physiological/MCAO conditions (Figure 1C,D,F). This indicated that PGRMC2 was mainly expressed in neuronal cells, astrocytes, and microglia under physiological conditions, and that its levels were elevated in neuronal cells and astrocytes, but not microglia following ischemic stroke. PGRMC2 showed no obvious co‐staining with CD31 under physiological conditions (Figure 1E); however, 3 days after MCAO, the levels of PGRMC2 and CD31 were significantly elevated, indicating that PGRMC2 was not directly expressed in endothelial cells under physiological condition. The elevated signals observed in endothelial cells will be addressed in a future article. Taken together, our data showed elevated PGRMC2 levels in different brain cells after ischemic brain injury.

### 3.2 CPAG‐1 penetrates the BBB and appears in both plasma and brain parenchyma after ischemic stroke

CPAG‐1 is a gain‐of‐function ligand of PGRMC2; however, the BBB permeability of CPAG‐1 is unknown. Thus, we intraperitoneally injected synthesized CPAG‐1 compounds immediately after the onset of MCAO, followed by 6, 12, and 24 h of reperfusion, and then measured CPAG‐1 concentrations by LC/MS. The molecular structure of CPAG‐1 and experimental design are shown in the Supplementary Information (Figure S1A,B). The concentration of CPAG‐1 increased to 942.4 ng/mL in plasma at 6 h after injection, decreased to 265.4 ng/mL at 12 h after injection, and decreased further to 127.68 ng/mL at 24 h after injection (Figure S1C). The concentration of CPAG‐1 in the brain parenchyma increased to 311.6 ng/g (contralateral) and 254.5 (ipsilateral) at 6 h after injection, decreased to 20.08 ng/g (contralateral) and 50.36 ng/g (ipsilateral) at 12 h after injection, and decreased further to 9.51 ng/g (contralateral) and 41.59 ng/g (ipsilateral) at 24 h after injection (Figure S1D,E). Taken together, these data indicated that the BBB is permeable to CPAG‐1.

### 3.3 CPAG‐1 treatment reduces infarct size and brain edema 3 days after ischemic stroke

**FIGURE 2:**
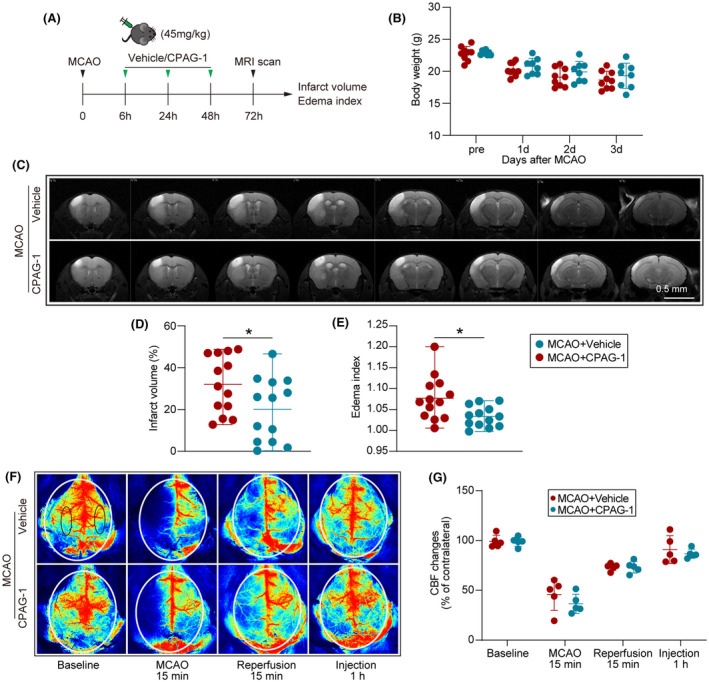
Intraperitoneal delivery of CPAG‐1 reduced brain infarction in mice 3 days after cerebral ischemia. Male C57BL/6J mice were subjected to MCAO and 3 days of reperfusion, and intraperitoneally treated with CPAG‐1 (45 mg/kg) or vehicle at 6, 24, and 48 h after the onset of MCAO. (A) Illustration of the experimental timeline. (B) Statistical analysis of body weight changes in CPAG‐1‐treated or vehicle‐treated mice after MCAO (*n* = 13/group, two‐way ANOVA, and Tukey's post‐hoc test). The infarct volume and brain edema index were examined using a 7.0 T MRI. (C) Representative MRI T2‐weighted images of CPAG‐1‐treated or vehicle‐treated mice 3 days after MCAO. (D) Statistical analysis of infarct volume and (E) edema index based on MRI (*n* = 13/group, one‐way ANOVA, and Tukey's post‐hoc test). Regional cerebral blood flow was examined using laser speckle imaging after CPAG‐1 or vehicle treatment. (F) Representative CBF images were obtained 15 min before MCAO (baseline), 15 min after the onset of MCAO (ischemia), 15 min after the onset of reperfusion (reperfusion), and 1 h after intraperitoneal injection of vehicle or CPAG‐1. Black circles: region of interest. (G) Quantitative analysis of regional CBF. Data are expressed as mean ± SD. *p < 0.05, **p < 0.01, *p < 0.001 versus MCAO + Vehicle group.

We investigated whether a gain‐of‐function of PGRMC2 could affect brain infarct volume and edema by intraperitoneal injection of 45 mg/kg of CPAG‐1 or vehicle at 6, 24, and 48 h after the onset of MCAO. The infarct size was examined by 7.0 T MRI 3 days after MCAO and treatment (Figure 2A). Intraperitoneal injection of CPAG‐1 had no significant effect on body weight at 1–3 days after MCAO compared with vehicle controls (Figure 2B). The infarct volumes were smaller, and the brain edema was less in the CPAG1‐treated mice than in the vehicle controls (Figure 2C‐E). Monitoring of the regional cerebral blood flow (rCBF) changes by laser speckle imaging (Figure 2F,G) revealed no significant differences in rCBF changes in the CPAG‐1‐treated mice and the vehicle controls at 15 min before MCAO, 15 min after MCAO, 15 min after reperfusion, or 1 h after injection.

### 3.4 CPAG‐1 treatment reduces BBB leakage and infiltration of peripheral neutrophils to brain parenchyma 24 h after ischemic stroke

**FIGURE 3:**
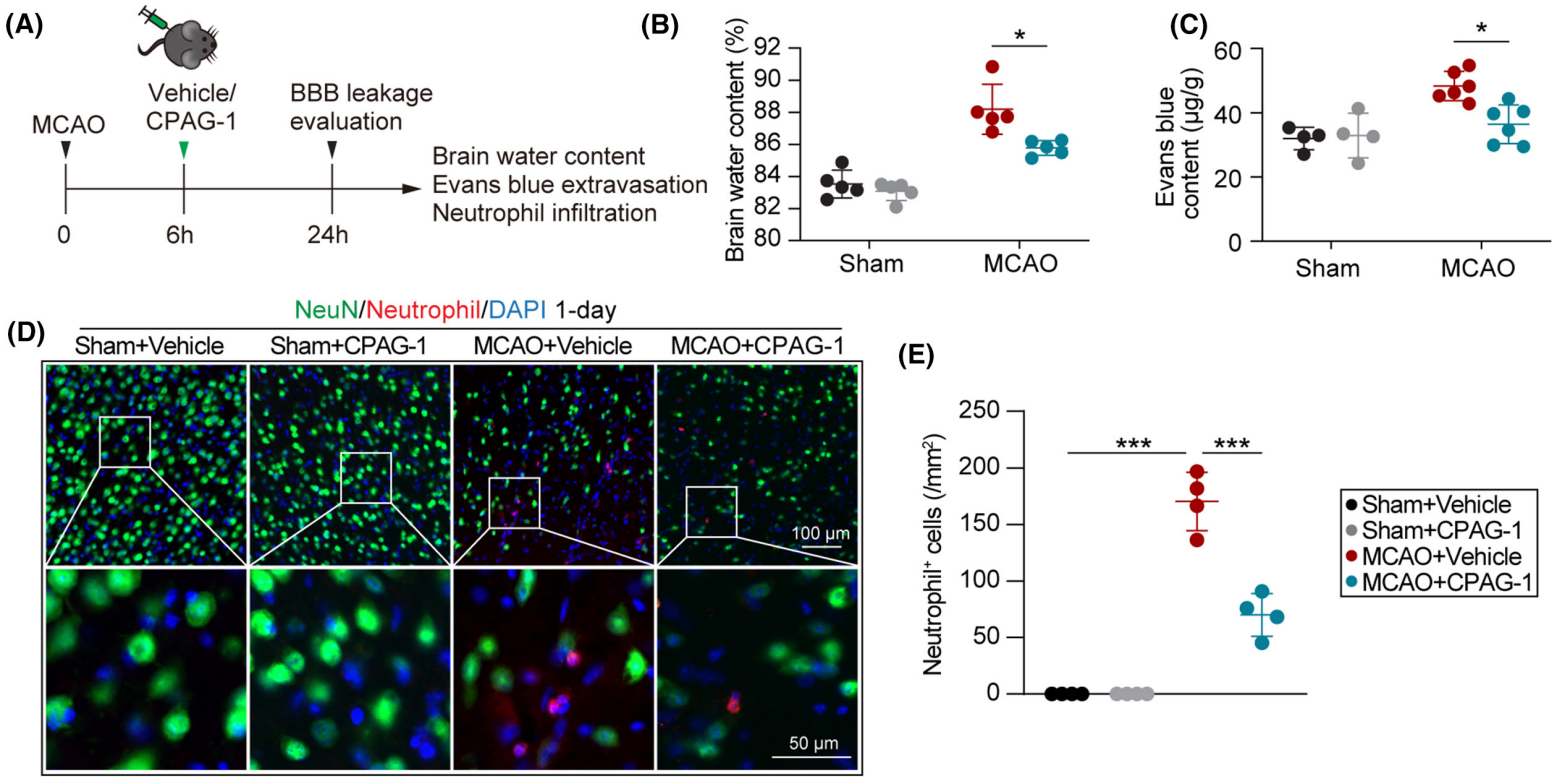
CPAG‐1 treatment reduces BBB leakage in mice 24 h after cerebral ischemia. Male C57BL/6J mice were subjected to either MCAO and 24 h of reperfusion or a sham operation, and then intraperitoneally injected with CPAG‐1 (45 mg/kg) or vehicle at 6 h after the operation. BBB leakage was evaluated by brain water content and Evans blue extravasation. (A) Illustration of the experimental timeline. (B) Brain water content. (C) Quantitative analysis of Evans blue content in the brain parenchyma. Infiltration by peripheral neutrophils was examined by immunostaining. (D) Representative images of NeuN (green)/Neutrophil (red) dual‐immunofluorescence staining, and (E) quantitative analysis. Data are presented as mean ± SD, *n* = 4‐6/group. Statistical analyses were performed by one‐way ANOVA with Tukey's post hoc test. *p < 0.05, **p < 0.01, and ***p < 0.001 versus MCAO + Vehicle group.

We also evaluated the effect of CPAG‐1 on BBB leakage after 24 h of ischemic stroke by brain water content, Evans blue extravasation, and infiltration of peripheral neutrophils (Figure 3A). As shown in Figure 3B, the brain water content was significantly lower in the CPAG‐1‐treated mice than in the vehicle controls (Figure 3B). No significant difference was detected in the brain water content between CPAG‐1‐ and vehicle‐treated sham mice (Figure 3B). Evans blue extravasation to ischemic brain parenchyma was significantly lower in CPAG‐1‐treated mice than in vehicle controls (Figure 3C). NeuN and neutrophil dual‐immunostaining to evaluate the infiltration of peripheral neutrophils after ischemic stroke (Figure 3D,E) revealed significantly greater numbers of neutrophil‐positive cells in peri‐infarct brain regions 1 day after operation in the MCAO mice than in the sham controls. Intraperitoneal injection of CPAG‐1 significantly reduced the number of neutrophil‐positive cells in the peri‐infarct brain regions, indicating that CPAG‐1 protects against BBB leakage after ischemic stroke in mice.

### 3.5 CPAG‐1 treatment ameliorates sensorimotor functions following ischemia

**FIGURE 4:**
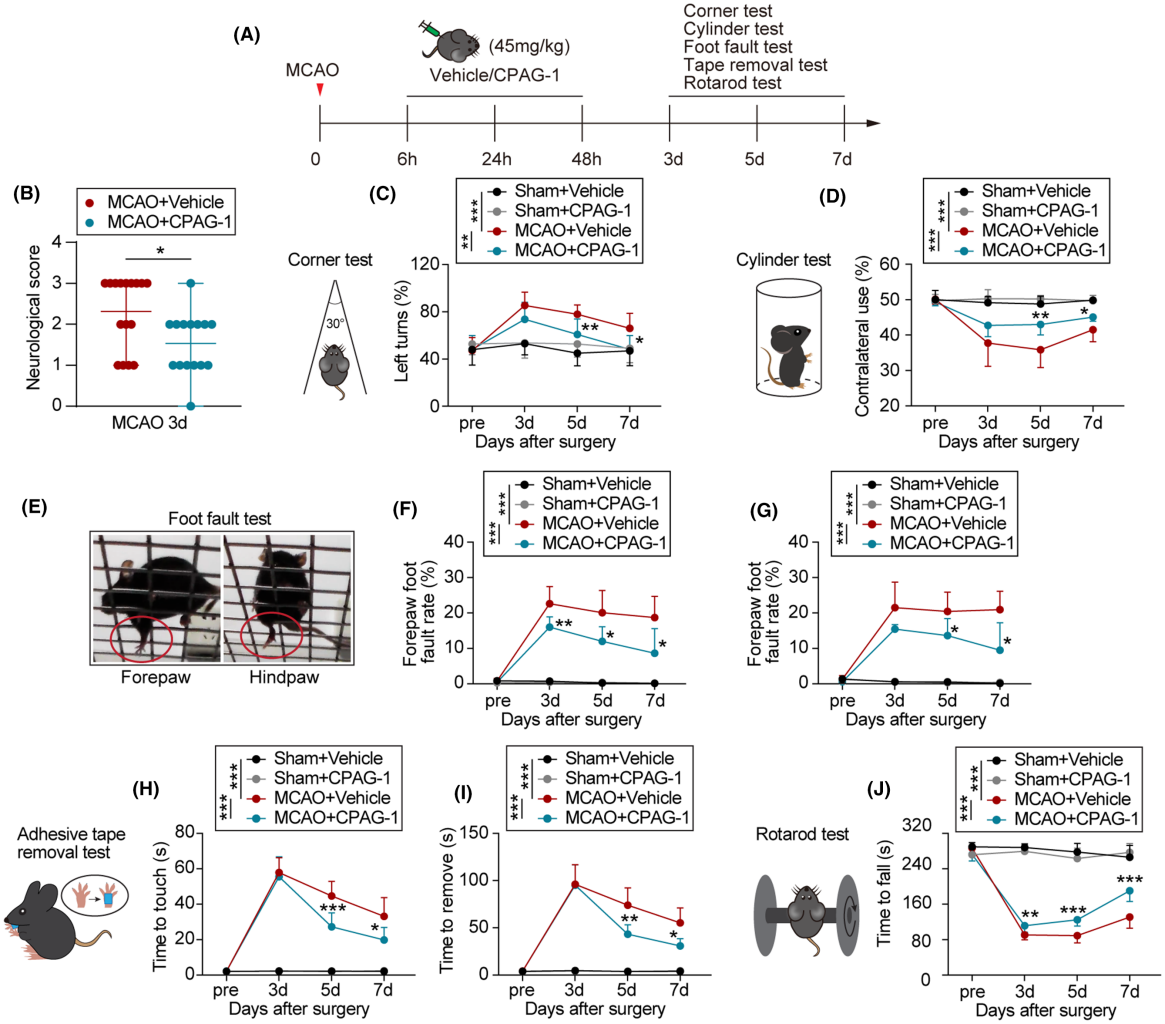
Intraperitoneal delivery of CPAG‐1 improves the acute sensorimotor functions in mice after ischemic stroke. Male C57BL/6J mice were subjected to either MCAO and up to 7 days of reperfusion or a sham operation, and were then intraperitoneally injected with CPAG‐1 (45 mg/kg) or vehicle at 6, 24, and 48 h after the operation. Sensorimotor functions were evaluated before and up to 7 days after MCAO by a panel of behavioral tests (*n* = 10–11/group) (A) Experimental design for behavioral evaluation. (B) Quantitative analysis of neurological deficits (*n* = 15–16/group, mean ± range, *t*‐test). (C) Left turns in the corner test. (D) Contralateral forepaw use in the cylinder test. (E) Representative images of forepaw and hindpaw foot faults. (F,G) Fault rates for the forepaw and hindpaw in the foot fault test. (H) Time to touch and (I) time to remove the tape in the adhesive tape removal test. (J) Time to fall in the rotarod test. Data are presented as mean ± SD, *n* = 10–11/group. Statistical analyses were performed by two‐way ANOVA with Tukey's post hoc test. *p < 0.05, **p < 0.01, and ***p < 0.001 versus MCAO + Vehicle group.

The panel of neurobehavioral tests, including corner, cylinder, adhesive tape removal, foot fault, and rotarod tests, were conducted to evaluate sensorimotor dysfunctions after 3–7 days of sham/MCAO operation and CPAG‐1/vehicle treatment (Figure 4A). The neurological scores were lower for mice receiving the CPAG‐1 treatment than for the vehicle controls (Figure 4B), suggesting that CPAG‐1 treatment improved neurological deficits. When compared with the sham controls, the MCAO mice showed severe sensorimotor dysfunctions 3–7 days after MCAO, as indicated by more left turns, less contralateral use, increased forepaw/hindpaw foot fault rate, more time to touch/remove the tapes, and less time to fall, as observed in the corner, cylinder, foot fault, adhesive tape removal, and rotarod tests, respectively (Figure 4C‐J). Sensorimotor performances were better in the CPAG‐1 mice than in the vehicle controls at 3–7 days after MCAO, as indicated by reduced times of left turns in the corner test (Figure 4C), increased contralateral forepaw use in the cylinder test (Figure 4D), reduced forepaw and hindpaw foot fault rate in the foot fault test (Figure 4E‐G), decreased time to touch and remove the adhesive tapes in the adhesive tape removal test (Figure 4H,I), and decreased time to fall in the rotarod test (Figure 4J). These data indicated that CPAG‐1 improved the sensorimotor dysfunctions associated with ischemic stroke.

### 3.6 Gene changes in response to CPAG‐1 treatment are primarily inflammation related

**FIGURE 5:**
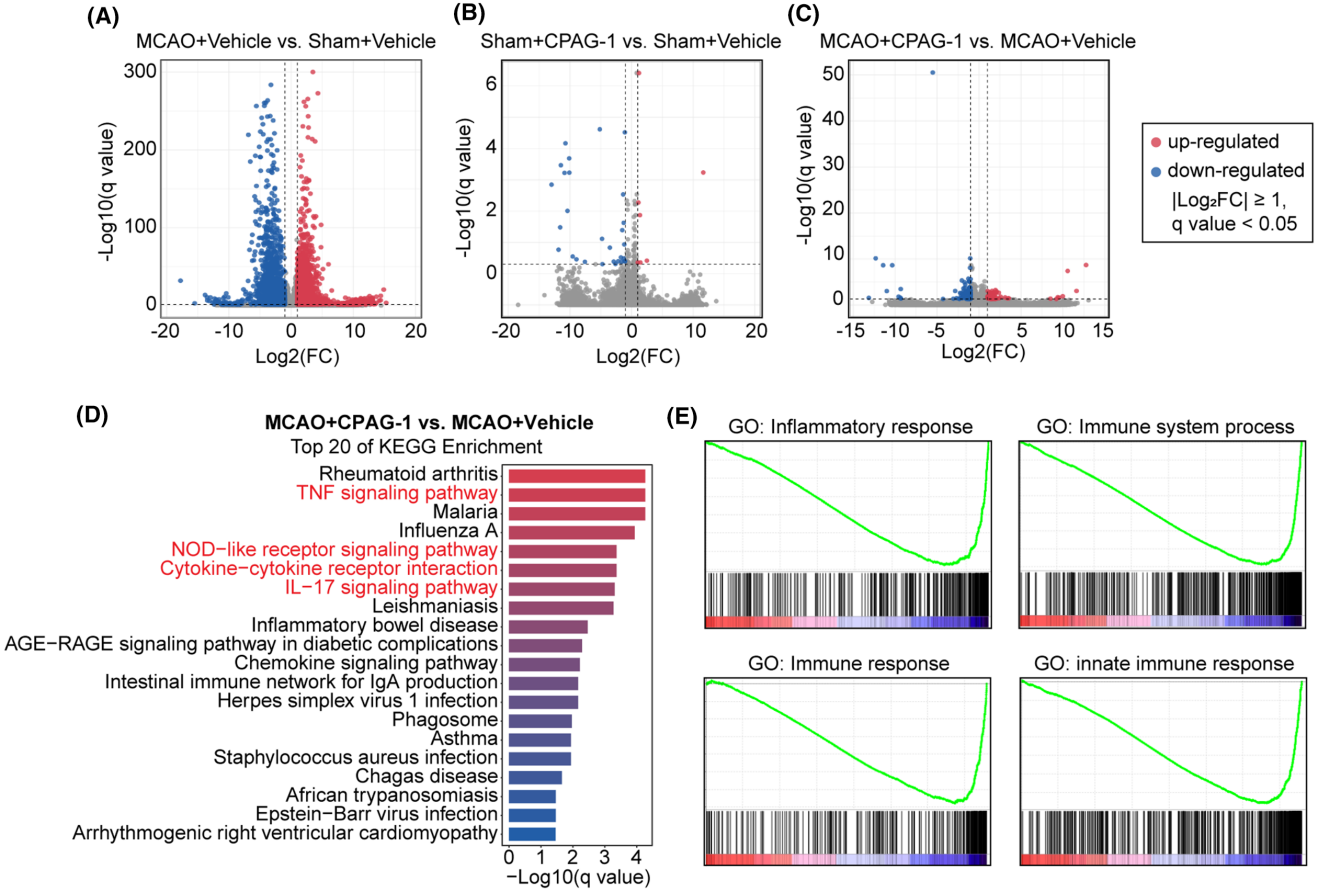
CPAG‐1 treatment alters mRNA profiles in mice 3 days after ischemic stroke. Male C57BL/6J mice were subjected to either MCAO and 3 days of reperfusion or a sham operation, and then intraperitoneally injected with CPAG‐1 (45 mg/kg) or vehicle at 6, 24, and 48 h after operation. The mRNA profiles were examined by RNA sequencing. Volcano plots demonstrate the differentially expressed genes (DEGs; |log_2_FC| ≥ 1, *q* value ≤ 0.05) in (A) MCAO+Vehicle versus Sham + Vehicle group, (B) Sham + CPAG‐1 versus Sham + Vehicle group, and (C) MCAO + CPAG‐1 versus MCAO + Vehicle group. (D) The top 20 enriched KEGG pathways in CPAG‐1‐treated mice compared with vehicle‐treated mice 3 days after MCAO. (E) Negatively enriched gene sets identified by GSEA.

We investigated the neuroprotective mechanisms underlying CPAG‐1 treatment by determining the mRNA expression profiles 3 days after MCAO in infarct core and peri‐infarct brain regions. As shown in Figure 5A, 2225 genes were upregulated and 1625 genes were downregulated 3 days after MCAO when compared with sham controls. Intraperitoneal delivery of CPAG‐1 upregulated 7 genes and downregulated 29 genes when compared with vehicle controls under sham conditions (Figure 5B). Intraperitoneal injection of CPAG‐1 in ischemic stroke mice upregulated 62 genes and downregulated 167 genes compared with vehicle controls (Figure 5C). KEGG pathway analysis revealed that, when compared with vehicle controls, the top 20 enriched pathways in CPAG‐1‐treated mice were mainly related to inflammation 3 days after ischemic stroke, and included the TNF signaling pathway, NOD‐like receptor signaling pathway, cytokine‐cytokine receptor interaction, and IL‐17 signaling pathway (Figure 5D). Gene set enrichment analysis (GSEA) from RNA‐sequencing data revealed negative regulation of gene sets related to inflammatory responses 3 days after MCAO in CPAG‐1‐treated mice compared with vehicle controls (Figure 5E). Taken together, these data indicated that the potential neuroprotective effect of CPAG‐1 might be associated with the suppressed expression of neuroinflammation genes after ischemic stroke.

### 3.7 CPAG‐1 treatment decreased astrocyte activation 3 days after ischemic stroke

**FIGURE 6:**
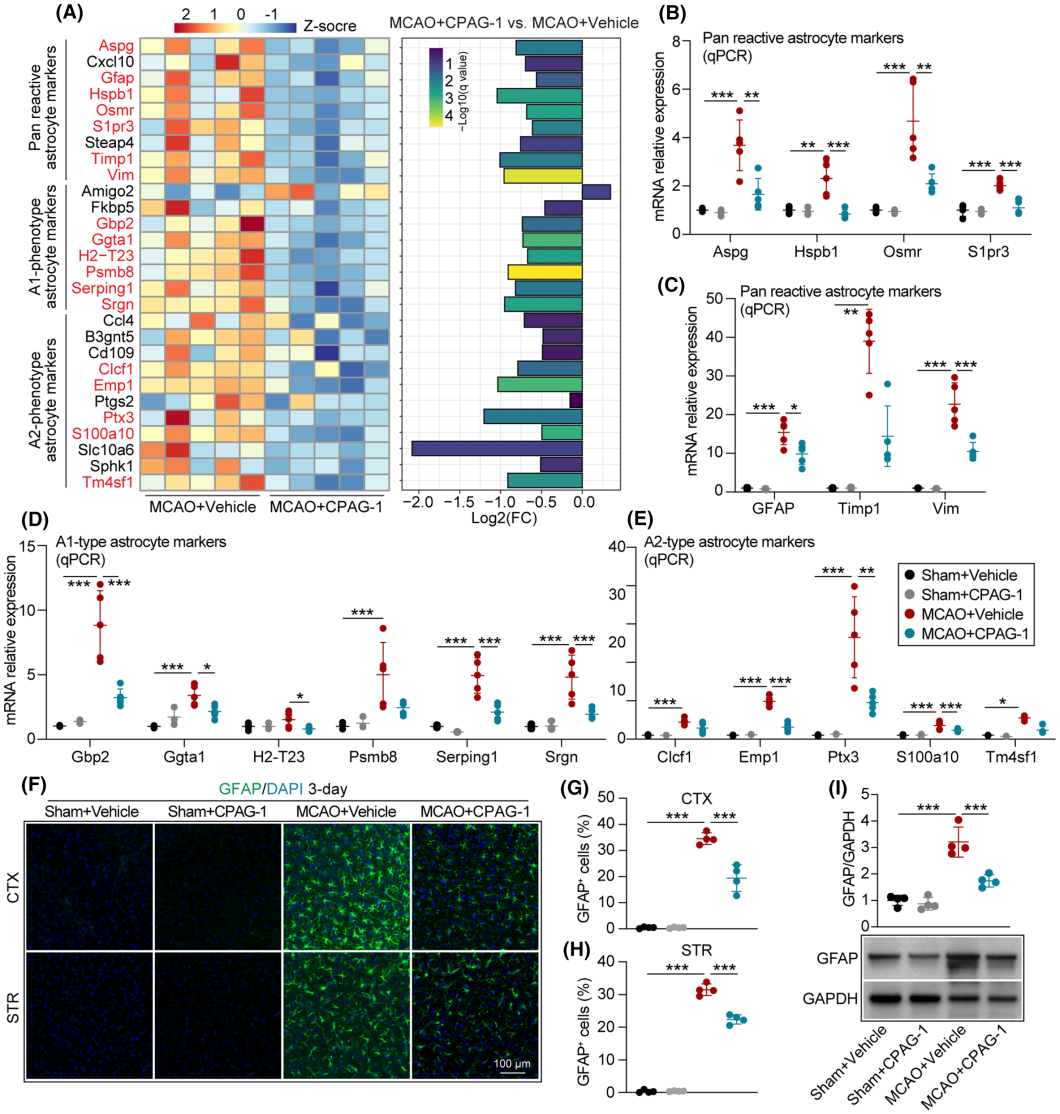
CPAG‐1 treatment reduces the astrocyte‐mediated inflammatory response in mice 3 days after ischemic stroke. Male C57BL/6J mice were subjected to either MCAO and 3 days of reperfusion or a sham operation, and intraperitoneally injected with CPAG‐1 (45 mg/kg) or vehicle at 6, 24, and 48 h after operation. The mRNA profiles of astrocyte‐related markers were analyzed using RNA‐sequence data. (A) The heatmap and fold changes of astrocyte‐related gene expression (red font: significantly changed genes, *q* value <0.05). The significantly altered genes from RNA‐sequence data were confirmed by qPCR. Quantitative analyses of mRNA expression of significantly altered (B,C) pan‐reactive astrocyte markers, (D) A1‐type astrocyte markers, and (E) A2‐type astrocyte markers. (F) Representative immunofluorescence staining of GFAP in the cerebral cortex and striatum (green). (G,H) Quantitative analysis of GFAP‐positive cells in the peri‐infarct cerebral cortex and striatum regions. (I) Representative western blotting images of GFAP and quantitative analysis. Data are presented as mean ± SD, *n* = 3‐5/group. Statistical analyses were performed by one‐way ANOVA with Tukey's post hoc test or the Kruskal–Wallis test. *p < 0.05, **p < 0.01, and ***p < 0.001 versus MCAO + Vehicle group.

We used the RNA‐sequence data to screen for altered expression in astrocyte‐related genes (fold changes and FPKM values), including pan‐reactive astrocyte markers, A1‐type (pro‐inflammatory type) astrocyte markers, and A2‐type (anti‐inflammatory type) markers (Figure 6A). Intraperitoneal delivery of CPAG‐1 significantly reduced the FPKM values of pan‐reactive, A1‐type, and A2‐type astrocyte markers 3 days after ischemic stroke when compared with vehicle controls. The figure shows significantly decreased genes (*q* value ≤ 0.05) marked in red font (Aspg, Gfap, Hspb1, Osmr, S1pr3, Timp1, Vim, Gbp2, Ggta1, H2‐T23, Psmb8, Serping1, Srgn, Clcf1, Emp1, Ptx3, S100a10, and Tm4sf1). Verification of the mRNA expression of significantly changed genes by qPCR revealed consistency with the RNA‐sequence results, except for several non‐significant altered genes, such as Timp1, Psmb8, Clcf1, and Tm4sf1 (Figure 6B‐E). Comparison of reactive astrocyte numbers in peri‐infarct brain regions between CPAG‐1 or vehicle‐treated mice 3 days after MCAO by GFAP immunostaining revealed increased numbers of GFAP‐stained cells in peri‐infarct cerebral cortex and striatum brain regions 3 days after MCAO when compared with sham controls, indicating robust astrocytosis in post‐stroke brains (Figure 6F‐H). Intraperitoneal delivery of CPAG‐1 reduced the numbers of GFAP‐stained cells in the peri‐infarct cerebral cortex and striatum brain regions 3 days after MCAO when compared with vehicle controls, suggesting that CPAG‐1 treatment suppressed astrocytosis in ischemic brains (Figure 6F‐H). The GFAP western blotting to quantify the GFAP protein expression also indicated that intraperitoneal injection of CPAG‐1 reduced the excessive astrocytosis in ischemic mouse brains, as suggested by less protein expression of GFAP in CPAG‐1‐treated mice than in vehicle controls (Figure 6I). These data indicated that CPAG‐1 is a robust suppressor of astrocyte activation.

### 3.8 CPAG‐1 treatment alters microglial polarization 3 days after ischemic stroke

**FIGURE 7:**
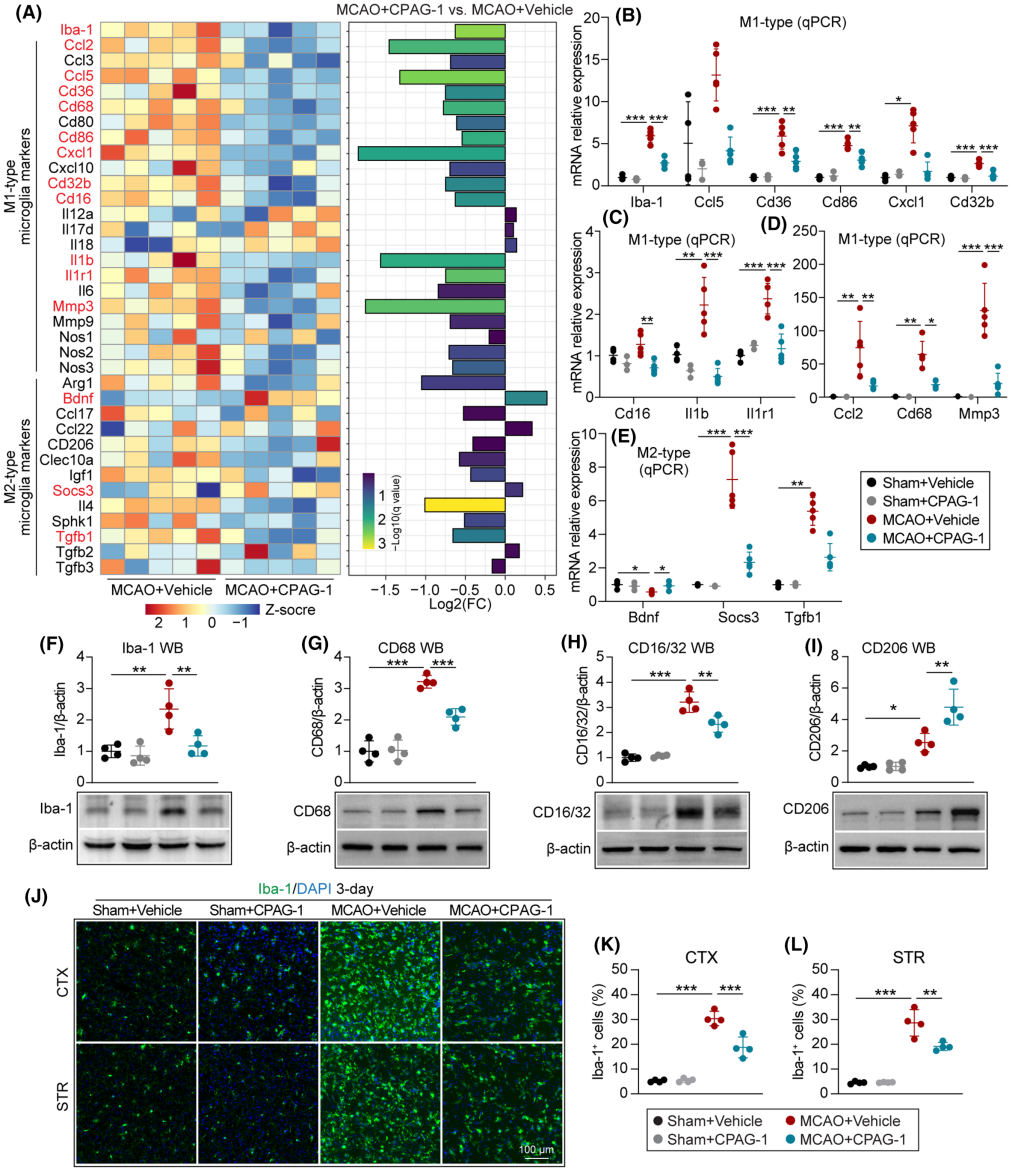
CPAG‐1 treatment alters microglial polarization in mice 3 days after ischemic stroke. Male C57BL/6J mice were subjected to either MCAO and 3 days of reperfusion or a sham operation, and then intraperitoneally injected with CPAG‐1 (45 mg/kg) or vehicle at 6, 24, and 48 h after operation. The mRNA profiles of microglia‐related markers were analyzed using RNA‐sequence data. (A) The heatmap and fold changes of microglia‐related gene expression (red font: significantly changed genes, *q* value ≤0.05). The significantly altered genes from RNA‐sequence data were confirmed by qPCR. Quantitative analyses of mRNA expression of significantly altered (B‐D) M1‐type microglia markers and (E) M2‐type microglia markers. (F‐I) Representative western blotting images and quantitative analysis of Iba‐1, CD68, CD86, and CD206. (J) Representative immunofluorescence images of Iba‐1 (green). (K,L) Quantitative analyses of Iba‐1‐positive cells in the peri‐infarct cerebral cortex and striatum regions. Data are presented as mean ± SD, *n* = 3‐5/group. Statistical analyses were performed by one‐way ANOVA with Tukey's post hoc test or the Kruskal–Wallis test. *p < 0.05, **p < 0.01, and ***p < 0.001 versus MCAO + Vehicle group.

Evaluation of the status of microglial polarization from RNA‐sequence data (Figure 7A) by analysis of the FPKM values and fold changes of microglial markers identified significantly changed genes (*q* value ≤0.05; marked in red font) between the MCAO+CPAG‐1 and MCAO + Vehicle groups. These significantly altered genes (marked in red font), which included pan‐reactive microglial marker (Iba‐1), M1‐type microglial markers (Ccl2, Ccl5, Cd36, Cd68, Cd86, Cxcl1, Cd32b, Cd16, Il1b, Il1r1, and Mmp3), and M2‐type microglial markers (Bdnf, Socs3, and Tgfb1), were also verified by qPCR. Intraperitoneal injection of CPAG‐1 significantly repressed the mRNA expression of Iba‐1, Cd36, Cd68, Cd32b, Cd16, Il1b, Il1r1, Ccl2, Cd68, Mmp3, and Socs3 markers 3 days after MCAO compared with vehicle controls (Figure 7B‐E). However, compared with vehicle administration, CPAG‐1 administration increased the expression of Bdnf mRNA 3 days after MCAO in mice. Western blotting confirmed the increased protein expression of Iba‐1, CD68, CD16/32, and CD206 3 days after MCAO compared with the sham controls (Figure 7F‐I). However, intraperitoneal injection of CPAG‐1 significantly reduced the protein expression of Iba‐1, CD68, and CD16/32 (pro‐inflammatory marker), whereas it increased the protein expression of CD206 (inflammatory‐resolving marker) 3 days after MCAO compared with vehicle controls. The western blot confirmed these findings. Microgliosis was also observed after Iba‐1 immunostaining (Figure 7J). Intraperitoneal injection of CPAG‐1 reduced the numbers of Iba‐1‐positive cells in the peri‐infarct cerebral cortex and striatum brain regions compared with vehicle controls 3 days after MCAO, indicating that CPAG‐1 can serve as a microglial mediator to reduce pro‐inflammatory microglial polarization and partially increase inflammatory‐resolving microglial polarization.

### 3.9 CPAG‐1 treatment reduces neuronal death and improves synaptic plasticity 3 days after ischemic stroke

**FIGURE 8:**
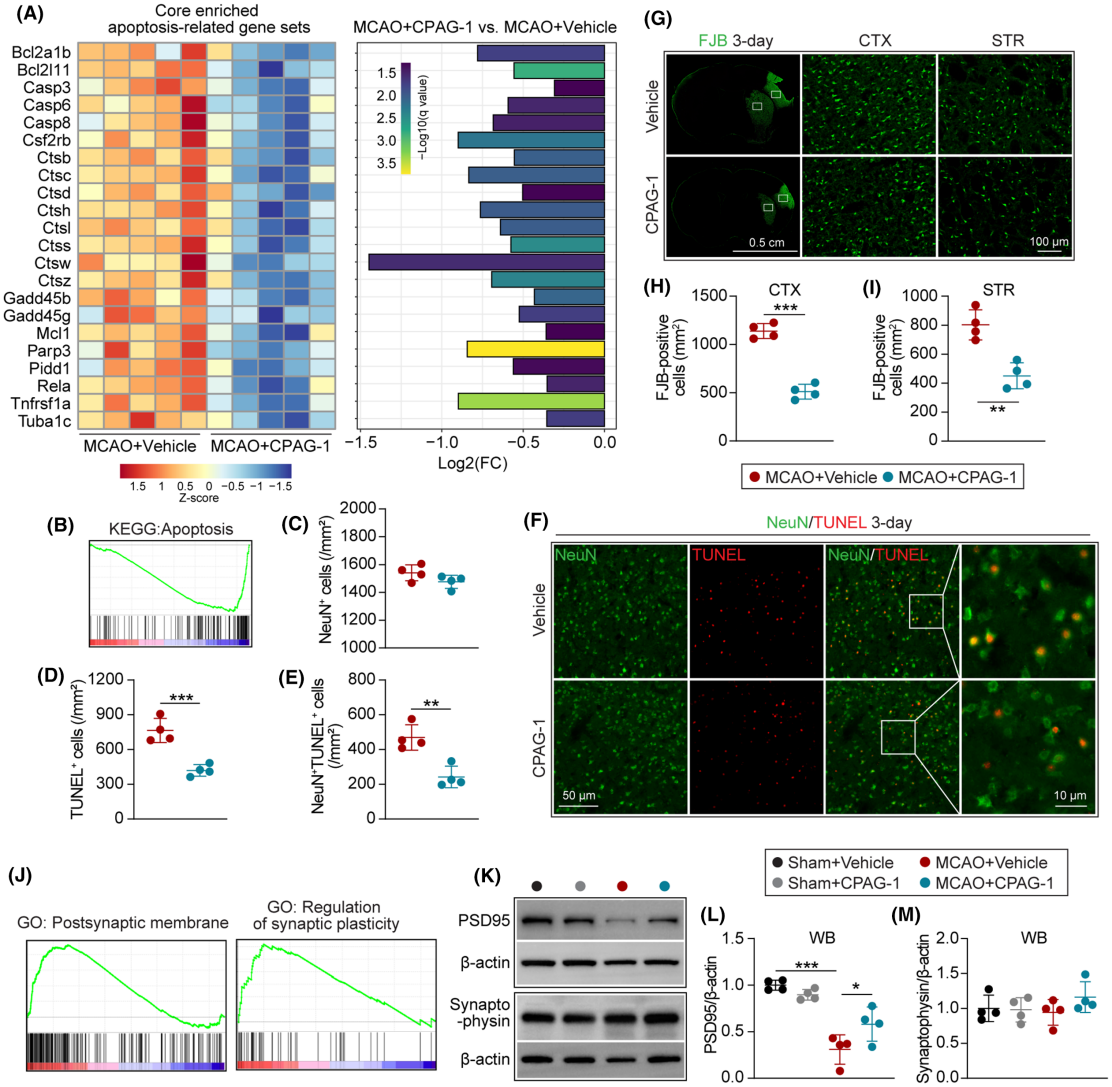
CPAG‐1 treatment reduces neuronal death and improves synaptic plasticity in mice after cerebral ischemia. Male C57BL/6J mice were subjected to either MCAO and 3 days of reperfusion or a sham operation, and intraperitoneally injected with CPAG‐1 (45 mg/kg) or vehicle at 6, 24, and 48 h after operation. The mRNA profiles of apoptosis‐related markers were analyzed from RNA‐sequence data. (A) The heatmap and fold changes of significantly altered core‐enriched apoptosis‐related gene sets. (B) Apoptosis‐enriched gene sets identified by GSEA. (C‐E) Quantitative analysis of NeuN^+^, TUNEL^+^, and NeuN^+^TUNEL^+^ cells in the peri‐infarcted brain regions. (F) Representative dual‐immunofluorescence staining of NeuN (green)/TUNEL (red). (G) Representative images of Fluoro‐Jade B (FJB) staining. (H,I) Quantitative analysis of FJB‐positive cells in the core‐infarcted cerebral cortex and striatum regions. (J) Synaptic plasticity‐enriched gene sets identified by GSEA. (K) Representative western blotting images of PSD‐95 and synaptophysin. (L,M) Quantitative analysis of PSD‐95 and synaptophysin. Data are presented as mean ± SD, *n* = 3‐5/group. Statistical analyses were performed by one‐way ANOVA with Tukey's post hoc test. *p < 0.05, **p < 0.01, and ***p < 0.001 versus MCAO + Vehicle group.

Gene set enrichment analysis showed that apoptosis‐related gene sets were significantly reduced in CPAG‐1‐treated mice 3 days after MCAO compared with vehicle controls (Figure 8B). The core‐enriched and significantly altered (*q* value ≤0.05) apoptosis‐related gene sets are shown in Figure 8A. NeuN/TUNEL dual‐immunostaining (Figure 8F) revealed that intraperitoneal injection of CPAG‐1 reduced the numbers of TUNEL‐positive cells and NeuN/TUNEL co‐stained cells in core‐infarct brain regions 3 days after MCAO when compared with vehicle controls (Figure 8D,E), indicating reduced neural and neuronal apoptosis after MCAO and CPAG‐1 treatment. The number of NeuN‐positive cells did not differ significantly between the experimental groups (Figure 8C). Examination of the degenerated neurons by FJB staining (Figure 8G) demonstrated that intraperitoneal injection of CPAG‐1 reduced the numbers of FJB‐positive cells in the core‐infarct cerebral cortex and striatum 3 days after MCAO when compared with vehicle controls (Figure 8H,I), indicating that CPAG‐1 treatment reduced neuron degeneration.

The GSEA results revealed that the gene sets positively enriched by CPAG‐1 treatment were related to synaptic plasticity. Western blotting for the synaptic plasticity‐related proteins PSD‐95 and synaptophysin (Figure 8K) confirmed a reduction in protein expression of PSD‐95 at 3 days after MCAO compared with sham controls, whereas intraperitoneal injection of CPAG‐1 increased the PSD‐95 expression 3 days after MCAO when compared with vehicle controls (Figure 8L). No significant difference was detected in synaptophysin expression among the experimental groups (Figure 8M). Taken together, these data suggested that CPAG‐1 preserves neuronal density and synaptic plasticity after ischemic stroke.

## 4 DISCUSSION

This study provides the first evidence that PGRMC2 is significantly elevated in different neural cells in the setting of ischemic stroke. We also achieved significant protective neuropathological and neurobehavioral effects by intraperitoneal injection of CPAG‐1, a gain‐of‐function ligand of PGRMC2, in our murine model of ischemic stroke.

Progesterone receptor membrane component 2 is widely expressed in urinary and reproductive systems, and the expression of PGRMC2 is altered in response to different pathophysiological conditions.^19^, ^21^, ^37^ In the brain, Intlekofer et al. found wide expression of mRNAs encoding classical and non‐classical progestin receptors, including PGRMC2, in the hippocampus, cerebral cortex, striatum, and other brain regions.^38^ PGRMC1 and PGRMC2 are non‐classical membrane‐associated progesterone receptors (MAPR). PGRMC1 and PGRMC2 possess an identical cytochrome b5‐heme/steroid binding domain (C‐terminus), and diverse N‐terminus and transmembrane domains. We previously reported significant elevations in the mRNA and protein levels of PGRMC1 in physiological and HIE mouse brains, as well as co‐expression of PGRMC1 in neuronal cells and astrocytes, except for microglia, in HIE brains.^24^ At present, the protein expression and cellular localization of PGRMC2 in brain tissue remain unexplored. The present findings demonstrate elevated protein expression of PGRMC2 in ischemic brains, with a predominant distribution in neuronal cells and astrocytes. The expression of PGRMC2 in microglia is low and did not show significant changes following ischemic stroke. Notably, PGRMC2 showed positive staining alongside the brain vessels. The accumulated signals around the brain vessels indicate a potential role for PGRMC2 in brain vessel functions that warrants further investigation.

CPAG‐1, which is a gain‐of‐function ligand of PGRMC2 was first synthesized by Parker and colleagues,^26^ who demonstrated that intraperitoneal injection of 45 mg/kg CPAG‐1 substantially improved diabetic symptoms. The BBB permeability of CPAG‐1 reported in the present study is a new finding. Our initial explorations of the distribution of CPAG‐1 in plasma and brain parenchyma after ischemic stroke in mice revealed a significant increase of CPAG‐1 in the plasma, contralateral hemisphere, and ipsilateral hemisphere after intraperitoneal injection of 45 mg/kg CPAG‐1 at 6–24 h after MCAO. Although we did not perform a detailed pharmacokinetics evaluation for CPAG‐1 in the present study, we at least confirmed the BBB permeability of CPAG‐1 following intraperitoneal injection, leading us to explore the potential neuroprotective effects of CPAG‐1 in the setting of ischemic stroke.

Previous studies have shown that progesterone administration and the activation of progesterone receptors protect against brain tissue loss and neurobehavioral deficits in several neurological disorders, such as traumatic brain injury, ischemic stroke, and HIE.^28^, ^39^, ^40^ Wali et al.^40^ demonstrated that intraperitoneal injection of moderate doses of progesterone reduced the infarct size and improved motor, sensory, and memory functions in a murine model of ischemic stroke over a large therapeutic time window. We previously demonstrated that intraperitoneal injection of progesterone in mice protected against cortical atrophy and improved the functional recovery of cognitive functions after HIE.^28^ We also reported that inhibition of PGRMC1 in mice increased brain tissue loss, sensorimotor deficits, and cognitive impairments after HIE.^24^ The regulatory role of PGRMC2 in neurological deficits remains poorly understood. The present findings are the first demonstration that the gain‐of‐function of PGRMC2 in mice can reduce brain infarction and acute sensorimotor deficits after ischemic stroke.

Blood–brain barrier disruption is a key feature of the acute phase of ischemic stroke.^6^ However, the importance of PGRMC2 in BBB maintenance has not been established. Previous studies have revealed that progesterone administration reduces the brain water content and Evans blue extravasation after ischemic brain injury.^40^, ^41^ We have previously shown that inhibition of PGRMC1 increased the brain water content in HIE mice.^24^ These findings suggest that progesterone signaling pathways may play a vital role in repairing BBB leakage. The present findings showed that the gain‐of‐function of PGRMC2 reduced Evans blue extravasation, brain edema, and brain water content that accompanies ischemic stroke. Disruption of the BBB structure and reduced expression or translocation of tight junction proteins from the membrane to the cytoplasm was followed by infiltration of peripheral immune cells, such as neutrophils and macrophages, into brain parenchyma and tremendous proinflammation. The gain‐of‐function of PGRMC2 reduced this infiltration of peripheral neutrophils into the ischemic brain parenchyma, suggesting that PGRMC2 gain‐of‐function protects against ischemic‐induced BBB disruption and might control the process of peripheral infiltration of immune cells. The detailed mechanisms underlying the BBB protection conferred by the CPAG‐1 administration require further investigation.

Suppressing excessive gliosis and controlling glial polarization are well‐recognized and vital therapeutic issues in the treatment of ischemic stroke. Resting astrocytes (A0) and microglia (M0) can be switched to their activated forms, either A1/M1 (neurotoxic) or A2/M2 (neuroprotective), in response to ischemic stroke.^42^ The A1‐type astrocyte and M1‐type microglia are viewed as causes of pro‐inflammatory activities, synaptogenesis loss, white matter damage, and neuronal death, whereas the A2‐type astrocytes and M2‐type microglia are inflammatory‐resolving and promote synaptic repair, growth, and neuronal survival.^8^, ^9^, ^42^, ^43^ The progesterone signaling pathway participates in the suppression of inflammation in neurological diseases, and progesterone treatment has been shown to inhibit astrocytosis, microgliosis, and oxidative stress after brain trauma in rodents.^39^, ^44^ Jiang et al.^45^ showed that intraperitoneal injection of progesterone suppressed TNF‐α levels in ischemic mouse brains, and we have consistently found that progesterone alleviates HIE‐induced brain injury by regulating the activation of TNF signaling.^28^ Bali et al.^46^ reported the expression of PGRMC1 in rat brain astrocytes and microglia and its regulation of their activations. By contrast, we did not detect any obvious expression of PGRMC1 in the mouse brain microglia.^24^ This difference in microglial expression might reflect differences in antibodies and species.

We have reported that the inhibition of PGRMC1 in HIE mouse brains decreased the levels of anti‐inflammatory mediators, including p‐AKT, p‐PI3K, and BDNF, and increased the expression of pro‐inflammatory mediators, including IL‐1α, IL‐1β, IL‐1R1, CCL‐2, IL‐6, p‐IKBα, and p‐NFκB. However, a role for PGRMC2 in astrocyte activation and microglia polarization has not been established. Our present findings showed that intraperitoneal injection of CPAG‐1 reduced inflammatory‐related gene sets, such as the TNF signaling pathway, NOD‐like receptor interaction, cytokine‐cytokine receptor pathway, and IL‐17 signaling pathway. Our screening of the astrocyte activation markers and microglial polarization markers after MCAO and CPGA‐1 treatment and subsequent RNA‐sequence and qPCR analysis showed that the gain‐of‐function of PGRMC2 suppressed pan‐reactive, A1‐type, and A2‐type astrocyte markers after ischemic injury. CPAG‐1 treatment also suppressed M1‐type microglia markers and several M2‐type microglia markers. The western blot and IF staining result also showed that CPAG‐1 treatment reduced astrocytosis and microgliosis, and switched the polarization of microglia from the pro‐inflammatory type (CD16/32) to the inflammatory‐resolving type (CD206). Interestingly, CPAG‐1 treatment increased the mRNA expression of BDNF, in agreement with other studies, after treatment with progesterone and a PGRMC1 inhibitor.^24^, ^47^ These results indicated a co‐regulation of BDNF by progesterone, PGRMC1, and PGRMC2. The underlying mechanism by which the progesterone signaling pathway co‐participates in the release of BDNF remains to be investigated in the future.

Progesterone and its receptors regulate the survival and synaptic plasticity of neurons. Sun et al. demonstrated that progesterone enhances the maturation and release of BDNF from glial cells and promotes synaptogenesis, as well as the survival of neurons in vitro. Nguyen et al.^48^ showed that inhibition of let‐7i, a negative upstream regulator of both PGRMC1 and BDNF in glia, has beneficial effects on neuronal viability and synaptogenesis in the ischemic brain. In our previous studies, we showed that intraperitoneal injection of progesterone reduced neuronal degeneration, while inhibition of PGRMC1 aggravated neuronal degeneration.^24^, ^28^ These results indicate that both progesterone and PGRMC1 have neuronal protection effects. However, the role of PGRMC2 in neuronal functions is unknown.

Our RNA‐sequence data uncovered significant repression of apoptosis‐related gene sets and an increase in synaptic plasticity‐related gene sets in ischemic mouse brains after CPAG‐1 treatment. Consistent with the RNA‐sequence results, we also observed reduced neuronal degeneration and apoptosis in CPAG‐1‐treated mouse brains after ischemic stroke. The CPAG‐1 treatment also preserved the synaptic plasticity‐related gene expression of synaptophysin but not of PSD‐95.

Collectively, the findings presented here are the first to support an elevated expression of PGRMC2 in different neural cells in the setting of cerebral ischemia. The intraperitoneal injection of CPAG‐1, a gain‐of‐function ligand of PGRMC2, conferred resistance to BBB leakage, astrocyte activation, microglial activation, neuronal death, and disruption of neuroplasticity while promoting the recovery of sensorimotor dysfunctions after ischemic stroke. These data support CPAG‐1 as a potential therapeutic candidate for brain repair.

## AUTHOR CONTRIBUTIONS

Conceptualization: FH, CZ; Methodology: CZ, TZ, HZ; Investigation: CZ, TZ, WN; Visualization: WN, CY, MW; Supervision: FH, GJ, JH, YZ; Writing—original draft: CZ; Writing—review & editing: FH, CZ. All authors read and approved the final manuscript.

## FUNDING INFORMATION

This work was supported by the National Nature Science Foundation of China to FH (81571469 and 82171420).

## CONFLICT OF INTEREST STATEMENT

The authors declare that they have no competing interests.

## Supporting information


AppendixS1

## Data Availability

The datasets used and/or analysed during the current study are available from the corresponding author on reasonable request.
