# Supplementary material for: Gain‐of‐function of progesterone receptor membrane component 2 ameliorates ischemic brain injury
Source: CNS Neurosci Ther. 2023 Feb 16;29(6):1585–601. doi: 10.1111/cns.14122 (PMC10173723; doi:10.1111/cns.14122)
Supplement: Supplementary file 1 — AppendixS1 [file CNS-29-1585-s001.docx]

**Supplementary Information**

**Gain-of-function of progesterone receptor membrane component 2 ameliorates ischemic brain injury**

Chao Zhou^1, 2, #^, Taiyang Zhu^1, 2, #^, Wanyan Ni^1, 2^ , Hui Zhou^1, 2^, Jiaxing Song^3^, Miao Wang^4^, Guoliang Jin^1, 2^, Yan Zhou^1, 2^, Jingjing Han^1, 2^, Fang Hua^1, 2, 5,^ *

1. Institute of Neurological Diseases, Xuzhou Medical University, Xuzhou, China, 221006

2. Department of Neurology, The Affiliated Hospital of Xuzhou Medical University, Xuzhou, China, 221006

3. Department of Neurology, Xinqiao Hospital and The Second Affiliated Hospital, Third Military Medical University, Chongqing, China, 400030

4. Department of Geriatrics, The Affiliated Hospital of Xuzhou Medical University, Xuzhou, China, 221006

5. Department of Interdisciplinary Health Science, College of Allied Health Science, Augusta University, USA, 30912

*Corresponding addressed to: Fang Hua, M.D., Ph.D.

Department of Neurology, The Affiliated Hospital, Xuzhou Medical University Xuzhou, Jiangsu 221002, China. Email: [huafang@xzhmu.edu.cn](mailto:huafang@xzhmu.edu.cn); fhua@augusta.edu

# The authors contributed equally to this work.

**Running title:** PGRMC2 activator ameliorates ischemic stroke

**
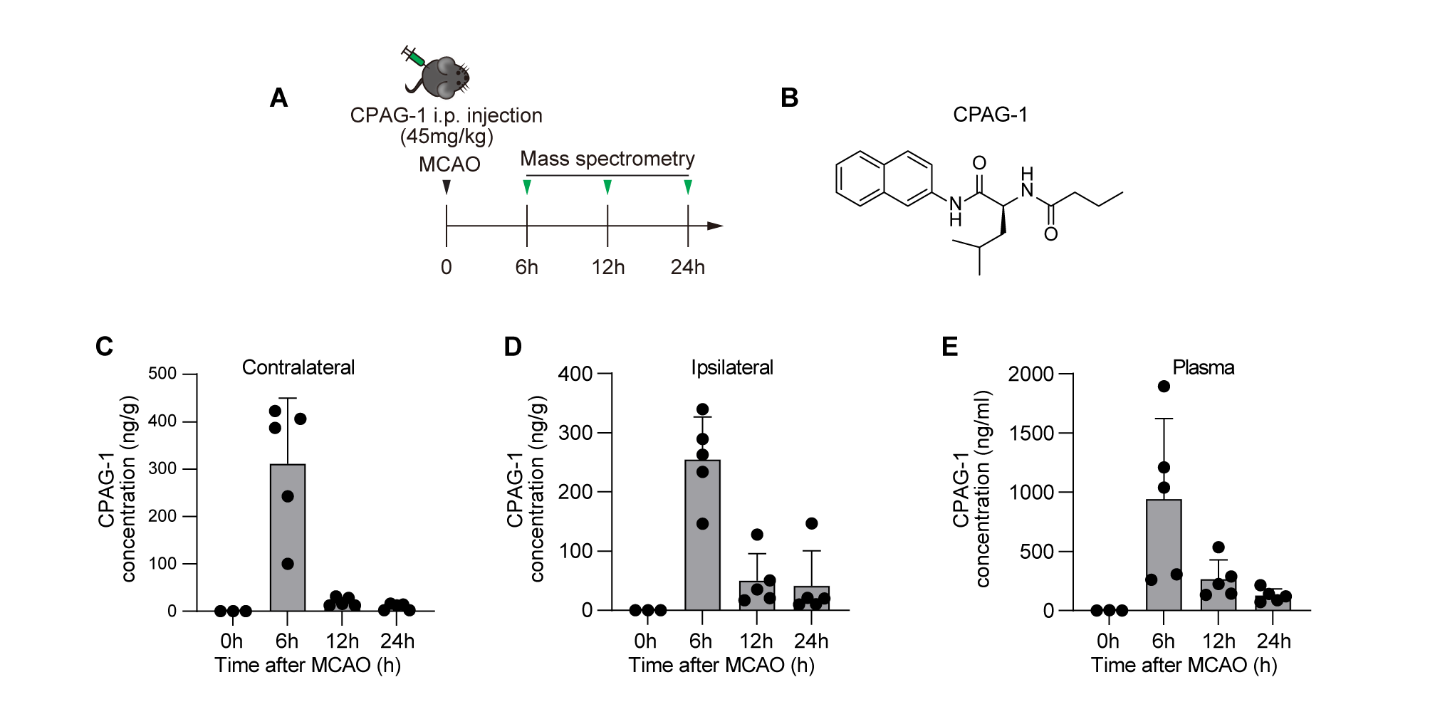
Figure S1: CPAG-1 distribution in plasma and brain parenchyma.** CPAG-1 was synthesized and intraperitoneally injected into mice immediately after the onset of MCAO and followed by 6 h, 12 h, and 24 h of reperfusion. The concentration of CPAG-1 was examined by LC/MS. **(A)** Experimental timeline. **(B)** Molecular structure of CPAG-1. **(C-E)** Statistical analysis of CPAG-1 distribution in contralateral/ipsilateral hemispheres, and plasma after ischemic stroke in mice.

**Table S1: List of primary antibodies used in this study.**

| **Antibody** | **Host species** | **WB dilution** | **IF stain dilution** | **Company** | **Cat #** |
| --- | --- | --- | --- | --- | --- |
| PGRMC2 | Mouse | 1:500 | 1:500 | Sigma-Aldrich | WH0010424M4 |
| CD31 | Rabbit |  | 1:200 | BD Biosciences | 553370 |
| NeuN | Rabbit |  | 1:1000 | EMD Millipore | ABN78 |
| GFAP | Rabbit | 1:500 | 1:1000 | Sigma-Aldrich | G9269 |
| Iba-1 | Rabbit |  | 1:500 | Wako | 019-19741 |
| Iba-1 | Mouse | 1:500 |  | HuaBio | RT1316 |
| CD68 | Rabbit | 1:500 |  | Proteintech | 28058-1-AP |
| CD16/32 | Rabbit | 1:200 |  | Invitrogen | MA5-29707 |
| CD206 | Rabbit | 1:500 |  | CST | 24595 |
| Neutrophil | Rat |  | 1:100 | Abcam | ab2557 |
| PSD-95 | Rabbit | 1:500 |  | Proteintech | 20665-1-AP |
| Synaptophysin | Rabbit | 1:500 |  | Proteintech | 17785-1-AP |
| β-actin | Mouse | 1:5000 |  | Proteintech | 66009-1-Ig |
| GAPDH | Mouse | 1:5000 |  | Proteintech | 60004-1-Ig |

**Table S2: List of primers used in this study.**

| **Genes** | **Primer sequences** |
| --- | --- |
| Aif1 | F: TCTGCCGTCCAAACTTGAAGCC; R: CTCTTCAGCTCTAGGTGGGTCT |
| Aspg | F: GCAATCGGACAACCAAGGTGGA; R: TACTGTGGACCACCAGATGGCT |
| Bdnf | F: GGCTGACACTTTTGAGCACGTC; R: CTCCAAAGGCACTTGACTGCTG |
| Ccl2 | F: GCTACAAGAGGATCACCAGCAG; R: GTCTGGACCCATTCCTTCTTGG |
| Ccl3 | F: ACTGCCTGCTGCTTCTCCTACA; R: ATGACACCTGGCTGGGAGCAAA |
| Ccl5 | F: ATATGGCTCGGACACCACTC; R: CTTCGAGTGACAAACACGACTG |
| CD36 | F: GGACATTGAGATTCTTTTCCTCTG; R: GCAAAGGCATTGGCTGGAAGAAC |
| CD68 | F: GGCGGTGGAATACAATGTGTCC R: AGCAGGTCAAGGTGAACAGCTG |
| CD86 | F: GACCGTTGTGTGTGTTCTGG; R: GATGAGCAGCATCACAAGGA |
| Clcf1 | F: GGAGCATCAACTCCGCAGCTTA; R: CCACACTTCCAAGTTGACCGTG |
| Cxcl1 | F: TCCAGAGCTTGAAGGTGTTGCC; R: AACCAAGGGAGCTTCAGGGTCA |
| Emp1 | F: TCCCTGTCCTACGGCAATGAAG; R: CTGGAACACGAAGACCACAAGG |
| Fcgr2b | F: CTACTGTGGACAGCCGTGCTAA; R: TCACCGTGTCTTCCTTGAGCAC |
| Fcgr3 | F: CAGAATGCACACTCTGGAAGC; R: GGGTCCCTTCGCACATCAG |
| Gbp2 | F: AGATGCCCACAGAAACCCTCCA; R: AAGGCATCTCGCTTGGCTACCA |
| Gfap | F: CACCTACAGGAAATTGCTGGAGG; R: CCACGATGTTCCTCTTGAGGTG |
| Ggta1 | F: GCTGATTGTCTCAACCGTGGTTG; R: CTGCCATCTGTTCTCACCAACC |
| H2-T23 | F: GTGGCTCCATAGATACCTACGG; R: GGTGATGTCAGCAGGGTAGAAG |
| Hspb1 | F: ATCACTGGCAAGCACGAAGA; R: GGCCTCGAAAGTAACCGGAA |
| Il1b | F: TGGACCTTCCAGGATGAGGACA; R: GTTCATCTCGGAGCCTGTAGTG |
| Il1r1 | F: CTGTTGGTGAGGAATGTGGCTG; R: GGCTCAGGATAACAGGTCTGTC |
| Il4 | F: ATCATCGGCATTTTGAACGAGGTC; R: R: ACCTTGGAAGCCCTACAGACGA |
| Il18 | F: GACAGCCTGTGTTCGAGGATATG; R: TGTTCTTACAGGAGAGGGTAGAC |
| Mmp3 | F: CTCTGGAACCTGAGACATCACC; R: AGGAGTCCTGAGAGATTTGCGC |
| Nos2 | F: GAGACAGGGAAGTCTGAAGCAC R: CCAGCAGTAGTTGCTCCTCTTC |
| Osmr | F: CCACTTCTGGAAATGGAGCGAC; R: ATGCGTCTTCCATTCTCCGACC |
| Psmb8 | F: CCTTACCTGCTTGGCACCATGT; R: TTGGATGCTGCAGACACGGAGA |
| Ptx3 | F: CGAAATAGACAATGGACTTCATCC, R: CATCTGCGAGTTCTCCAGCATG |
| S1pr3 | F: GCTTCATCGTCTTGGAGAACCTG; R: CAGAGAGCCAAGTTGCCGATGA |
| Serping1 | F: TTGCCTGTGTCCACCAAGCACT; R: GCTGCTTCCATACAGGCTCTGA |
| Socs3 | F: GGACCAAGAACCTACGCATCCA; R: CACCAGCTTGAGTACACAGTCG |
| Srgn | F: TGCGAACTGCATCGAGGAGAAG R: CCCGAACCTGACCCATAGTCAT |
| S100a10 | F: GACAAAGGAGGACCTGAGAGTG R: CTCTGGAAGCCCACTTTGCCAT |
| Tgfb1 | F: TGATACGCCTGAGTGGCTGTCT; R: CACAAGAGCAGTGAGCGCTGAA |
| Timp1 | F: TCTTGGTTCCCTGGCGTACTCT; R: GTGAGTGTCACTCTCCAGTTTGC |
| Tm4sf1 | F: GGATGAAGAGGACTGCTGTGGT; R: CCACGATTCCAATCAGAGCAGC |
| Vim | F: CGGAAAGTGGAATCCTTGCAGG; R: AGCAGTGAGGTCAGGCTTGGAA |

**Table S3: Animal used and mortality of this study.**

| **Endpoint** | **Group** | **Survival** | **Total** | **Experiment** | **Mortality (%)** |
| --- | --- | --- | --- | --- | --- |
| 1 day | Sham+Vehicle | 12 | 12 | Brain water content  Evans blue  Neutrophil/NeuN stain | 0.00 |
|  | Sham+CPAG-1 | 12 | 12 |  | 0.00 |
|  | MCAO+Vehicle | 12 | 12 |  | 0.00 |
|  | MCAO+CPAG-1 | 12 | 12 |  | 0.00 |
| 3 days | Sham+Vehicle | 8 | 8 | MRI; CBF measurement  Body weight changes  IF stain and IHC stain  RNA-sequencing and qPCR | 0.00 |
|  | Sham+CPAG-1 | 8 | 8 |  | 0.00 |
|  | MCAO+Vehicle | 13 | 20 |  | 35.00 |
|  | MCAO+CPAG-1 | 13 | 19 |  | 30.00 |
| 1/3/7 days | Sham | 4 | 4 | PGRMC2 expression  (WB, IF stain) | 0.00 |
|  | MCAO | 15 | 20 |  | 25.00 |
| 0/6/12/24 h | MCAO | 18 | 20 | CPAG-1 LC/MS | 10.00 |
| 7 days | Sham+Vehicle | 10 | 10 | Neurobehavioral tests  Neurological deficits | 0.00 |
|  | Sham+CPAG-1 | 11 | 11 |  | 0.00 |
|  | MCAO+Vehicle | 10 | 14 |  | 28.57 |
|  | MCAO+CPAG-1 | 11 | 14 |  | 21.42 |
|  | Total | 169 | 196 |  |  |
